# Supplementary material for: Interlayer magnetophononic coupling in MnBi2Te4
Source: Nat Commun. 2022 Apr 8;13:1929. doi: 10.1038/s41467-022-29545-5 (PMC8993894; doi:10.1038/s41467-022-29545-5)
Supplement: Supplementary file 1 — Supplementary Information [file 41467_2022_29545_MOESM1_ESM.pdf]

Supplementary Information: Interlayer magnetophononic coupling in $MnBi_2Te_4$

Supplementary Note 1: Raman peak assignment and eigenvectors

Supplementary Note 2: Anomalous temperature-dependence of $A_{1g}^{(1)}$ mode

Supplementary Note 3: Field-dependence of $E_g^{(2)}$, $E_g^{(3)}$, and $A_{1g}^{(3)}$ spectral weights

Supplementary Note 4: Generalized magnetophononic coupling and frequency renormalization

Supplementary Note 5: Resonant Raman effects

Supplementary Note 6: Symmetry of anomalous scattering intensity

Supplementary Note 7: Pump-probe measurements – Fluence-dependence

Supplementary Note 8: Debye-Waller effect in ultrafast electron diffraction

Supplementary Table 1. Raman phonon mode assignment.

Supplementary Figure 1. Polarized Raman spectra

Supplementary Figure 2. Anomalous temperature-dependence of $A_{1g}^{(1)}$ amplitude.

Supplementary Figure 3. Absence of magnetophononic coupling.

Supplementary Figure 4. Spin-induced phonon frequency renormalization.

Supplementary Figure 5. Phonon spectra at different laser excitation energies.

Supplementary Figure 6. Temperature-dependent intensity of $A_{1g}^{(2)}$ mode with 1.58 eV excitation.

Supplementary Figure 7. Temperature- and polarization-dependent $A_{1g}^{(2)}$ scattering intensity.

Supplementary Figure 8. Fluence-dependence of coherent phonons.

Supplementary Figure 9. Transient Debye-Waller effect.

Supplementary Note 1: Raman peak assignment and eigenvectors

We start with a systematic analysis of Raman phonon spectra, shown in Supplementary Figure 1a. The non-magnetic unit cell contains seven atoms, and thus there are 21 phonon modes in total, consisting of 18 optical and 3 acoustic modes. Using representation theory, these can be decomposed into irreps of the point group $\bar{3}m$. Of these, only the E_g and A_{1g} modes are Raman active. Polarized Raman spectroscopy measurements are used to readily identify these modes based on their different selection rules. In particular, E_g modes have non-vanishing diagonal Raman tensor components and are thus visible under both parallel- and cross-polarized configurations, whereas the A_{1g} modes have only diagonal Raman tensor components and are visible only under the parallel-polarized configuration. We did not observe any dependence on the in-plane crystallographic orientation.

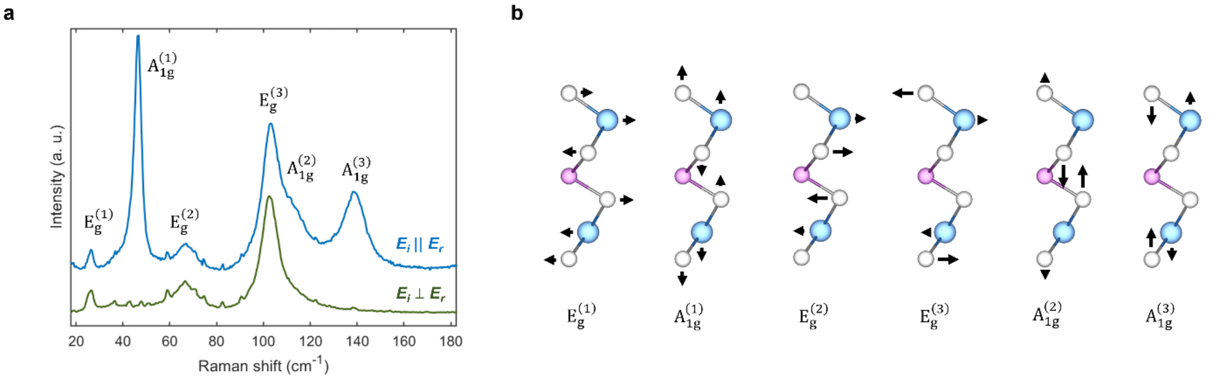

Supplementary Figure 1. Polarized Raman spectra. a, Raman spectra with the incident and reflected beams parallel- and cross-polarized with respect to each other, at 298 K. **b,** Eigenvectors of Raman phonons, with the arrows denoting ionic motions, calculated using density functional theory simulations. The arrow lengths are proportional to the actual calculated ionic eigendisplacements for all modes.

First-principles calculations are used to enumerate all the Γ-point Raman-active optical phonon modes and their energies in Supplementary Table 1. Good agreement is obtained between the calculations and measurements for all the observed Raman phonons, confirming that the first-principles calculations provide a good description of the lattice dynamics.

**Symmetry**	**Frequency (cm^{-1}) (theory, DFT)**	**Frequency (cm^{-1}) (experiment at 15 K)**
E_g	32.9	27.2
A_{1g}	50.7	49.1
E_g	79.8	69.8
E_g	112.9	108.3
A_{1g}	119.2	113.1
A_{1g}	148.9	146.6

Supplementary Table 1. Raman phonon mode assignment. Raman phonon symmetries and frequencies at the Γ point, from density functional theory calculations (theory), and Raman spectroscopy (experiment) at 15 K.

The phonon eigendisplacements at the Γ point, calculated using density functional theory simulations, show that A_{1g} phonons have purely out-of-plane ionic motions, whereas E_g phonons have purely in-plane ionic motions.

Supplementary Note 2: Anomalous temperature-dependence of $A_{1g}^{(1)}$ mode

In Supplementary Figure 2a, we plot the Raman spectra measured at 15 K and 300 K, normalized to the height of the $E_g^{(3)}$ peak at ~113 cm^{-1}, for convenience. We note that the result identified below is independent of the choice of normalization. In general, phonon peaks in Raman spectra broaden with increasing temperature due to increased phonon-phonon scattering, with resultant lower peak heights. This is visible for instance in the $A_{1g}^{(3)}$ peak at ~145 cm^{-1}. On the other hand, the scattering intensity of the $A_{1g}^{(1)}$ mode exhibits an anomalous temperature-dependence, with a dramatic *decrease* in height and integrated intensity, with decreasing temperature (see Supplementary Figure 2b). It is apparent that this decrease in amplitude is independent of the choice of normalization. The amplitude does not show any clear correlation with the magnetic transition at T_N = 24 K.

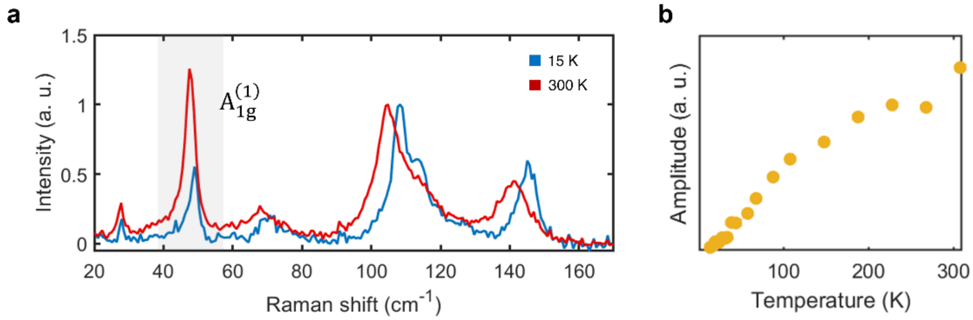

Supplementary Figure 2. Anomalous temperature-dependence of $A_{1g}^{(1)}$ amplitude. a, Unpolarized Raman spectra at 15 K and 300 K, normalized to the height of the $E_g^{(3)}$ peak at ~115 cm^{-1}. The $A_{1g}^{(1)}$ mode is highlighted in grey. **b,** The amplitude of the $A_{1g}^{(1)}$ peak, fit to a Fano lineshape, as outlined in the Methods section.

A possible explanation for the dramatic change in scattering intensity with temperature is proximity of the Raman excitation energy (633 nm = 1.96 eV) to electronic transitions correlated with the ionic motion of the $A_{1g}^{(1)}$ mode. Optical conductivity measurements[1] indeed show large changes in the measured temperature range. Such resonant effects may be probed by measuring relative Raman phonon scattering cross-sections as a function of the excitation energy. Resonant effects are discussed in detail in Supplementary Note 5.

The scattering intensity associated with the zone-boundary $A_{1g}^{(1)}$ mode is clearly visible in the field-dependent Raman spectra in Fig. 1d in the main text. In the temperature-dependent Raman spectra in Fig. 1c however, the anomalous temperature-dependence, described above, appears to swamp the small zone-boundary scattering intensity.

Supplementary Note 3: Field-dependence of $E_g^{(2)}$, $E_g^{(3)}$, and $A_{1g}^{(3)}$ spectral weights

The integrated intensities of the $E_g^{(2)}$, $E_g^{(3)}$, and $A_{1g}^{(3)}$ phonons are plotted in Supplementary Figure 3a-c, respectively, as a function of magnetic field. The integrated intensities were obtained by fitting individual spectra following the procedure outlined in the Methods section, with the error bars denoting the standard deviation in fit values. We note a small dip in the $E_g^{(2)}$ intensity at the spin-flop critical field of 3.7 T. Outside of this, the three modes shown here exhibit no clear field-dependent behavior above the experimental and fitting uncertainty. In particular, there is no signature of coupling to the antiferromagnetic order parameter and the associated zone-boundary phonons.

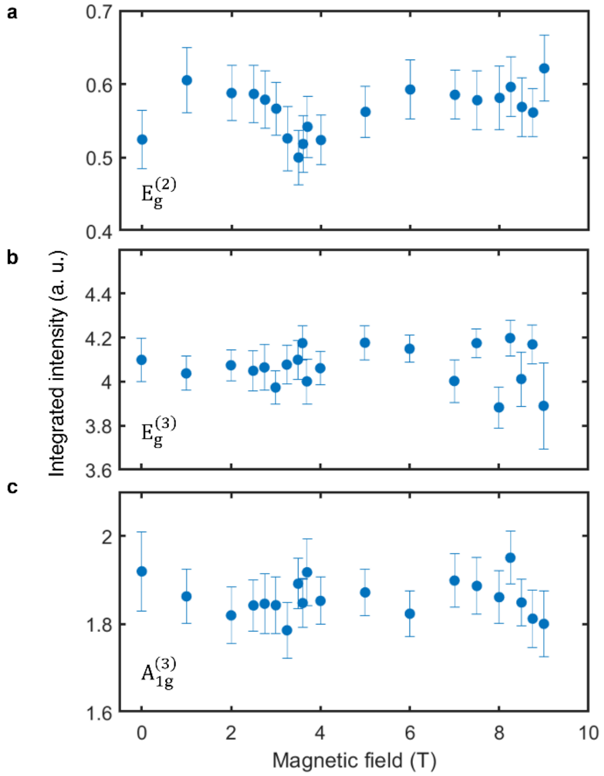

Supplementary Figure 3. Absence of magnetophononic coupling. The panels show spectral weights of the **a,** $E_g^{(2)}$, **b,** $E_g^{(3)}$, and **c,** $A_{1g}^{(3)}$ modes respectively, as a function of magnetic field. The error bars are standard deviations of the fit values.

Supplementary Note 4: Generalized magnetophononic coupling and frequency renormalization

We write down minimal lattice and spin Hamiltonians[2] to describe a generalized magnetophononic coupling. Consider the lattice Hamiltonian described by the harmonic approximation,

$$H_L = H_L^0 + \frac{1}{2!}\frac{\partial^2 H_L^0}{\partial u_\alpha^2}u_\alpha^2 + O(u_\alpha^3) \approx H_L^0 + \frac{1}{2}N\mu_\alpha {\nu_\alpha^0}^2 u_\alpha^2, \quad (1)$$

where u_α is the displacement along the phonon normal mode α, μ_α is the reduced mass, ν_α is the frequency, and N is the number of unit cells. The magnetic ground state energy described by a Heisenberg-like Hamiltonian,

$$H_M^0 = -\sum_{ij} J_{ij} S_i . S_j, \quad (2)$$

where i and j are spin site indices, and J_{ij} is the isotropic exchange interaction between spins at i and j. When this is perturbed by a zone-center optical phonon α, the perturbed magnetic energy can be derived by considering the derivatives of J_{ij} with respect to the phonon normal mode displacement u_α. Expanding upto second order in u_α , the perturbed exchange interaction is

$$J'_{ij}(u_\alpha) = J_{ij} + \frac{\partial J_{ij}}{\partial u_\alpha}u_\alpha + \frac{1}{2}\frac{\partial^2 J_{ij}}{\partial u_\alpha^2}u_\alpha^2. \quad (3)$$

Here, the first order term, in the specific case of $J = J^\perp$ is responsible for the magnetophononic wave-mixing described in detail in the main text (Supplementary Eq. 1 and Eq. 2).

The second order term, proportional to u_α^2, renormalizes the harmonic term in the lattice energy, resulting in spin-induced phonon frequency changes. Separating the in-plane and out-of-plane exchange couplings, denoted by J_μ and $J_\mu^\perp$, respectively, where $\mu = 1, 2, \ldots$ are the first- and second-nearest-neighbors and so on, and assuming small spin-induced energy shifts i. e. $\nu_\alpha + \nu_{\alpha 0} \approx 2\nu_{\alpha 0}$, the renormalized phonon frequency is given by

$$\nu_\alpha - \nu_{\alpha 0} = \frac{1}{4N\mu_\alpha \nu_{\alpha 0}}\left[\sum_\mu \frac{\partial^2 J_\mu}{\partial u_\alpha^2}\sum_i S_i . S_{i+\mu} + \sum_\mu \frac{\partial^2 J_\mu^\perp}{\partial u_\alpha^2}\sum_i S_i . S_{i+\mu^\perp}\right]. \quad (4)$$

The above expression shows the renormalization of the phonon frequency due to spin order along different directions, through the respective exchange couplings. Under a mean-field approximation, Supplementary Eq. 4 simplifies to $\nu_\alpha - \nu_{\alpha 0} \propto \langle S^2 \rangle$.

Experimentally, we observe such a spin-induced phonon frequency renormalization in the $A_{1g}^{(1)}$ mode. The phonon frequencies are first extracted as a function of temperature, using the fitting procedure outlined in the Methods section of the main text. We then account for phonon-phonon interactions by fitting the temperature-dependent phonon frequencies to that of a (cubic) anharmonic phonon, given by $\omega(T) = \omega_0 + A\left(1 + \frac{2}{e^{\frac{\hbar\omega_0}{2k_BT}} - 1}\right)$, where ω is the phonon frequency renormalized by anharmonic (phonon-phonon) interactions, T is the temperature, ω_0 is the bare phonon frequency, and A is a mode-specific fitting constant.

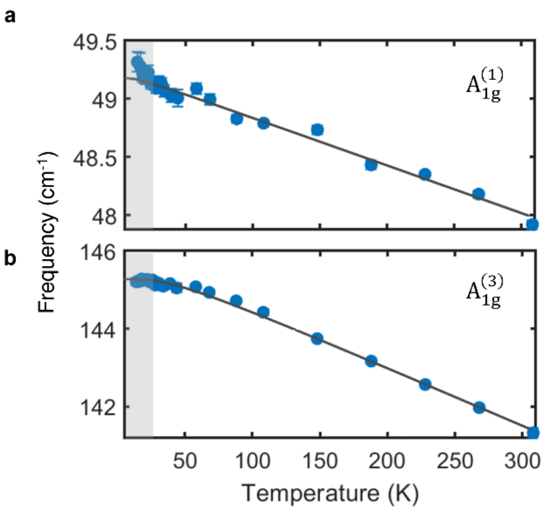

Supplementary Figure 4. Spin-induced phonon frequency renormalization. Temperature-dependent frequency of the $A_{1g}^{(1)}$ phonon mode (**a**) and $A_{1g}^{(3)}$ phonon mode (**b**). The black lines are fits to the anharmonic phonon model described in the text. Error bars are standard deviations in fit values.

Supplementary Figure 4 shows the temperature-dependent frequency of the $A_{1g}^{(1)}$mode, with the black line showing a fit to the anharmonic phonon model. The plot shows a small but clear deviation from the fit below $T_N = 24$ K, indicating a spin-induced phonon frequency renormalization. In contrast, the temperature-dependent frequency of the $A_{1g}^{(3)}$ mode in Supplementary Figure 3 shows good agreement with the anharmonic phonon model down to the lowest temperatures, indicating that the $A_{1g}^{(3)}$ mode does not exhibit a significant spin-induced frequency renormalization.

Interestingly, we note that a previous study[3] on atomically thin flakes of $MnBi_2Te_4$ reported a negative spin-induced frequency renormalization of the $A_{1g}^{(1)}$ mode, contrary to the positive frequency renormalization observed in the bulk crystals used in our study. This difference may possibly be due to changes in the electronic and magnetic structure as a function of sample thickness in the 2D limit.

The strong magnetophononic coupling observed in the $A_{1g}^{(2)}$ mode in our magneto-Raman measurements suggests that it too might exhibit a significant spin-induced frequency shift. Unfortunately, the spectral overlap between the $A_{1g}^{(2)}$ and $E_g^{(3)}$ modes (see Supplementary Figure 1a) and strong $A_{1g}^{(2)}$ zone-boundary scattering intensity below T_N hinders a similar temperature-dependent frequency analysis for the $A_{1g}^{(2)}$ mode. None of the other observed Raman phonons exhibit a spin-induced frequency renormalization above the experimental uncertainty.

Supplementary Note 5: Resonant Raman effects

Resonant Raman effects may potentially give rise to temperature- and field-dependent artifacts in phonon peak intensities due to changes in the electronic band structure across phase transitions. In order to rule out such an explanation for the phenomena reported in Fig. 1 and Fig. 2, we investigate resonant Raman effects in $MnBi_2Te_4$ by measuring phonon spectra at different laser excitation energies. In Supplementary Figure 5, we show the Raman phonon spectra measured with laser excitation energies of 1.58 eV (785 nm), 1.96 eV (633 nm), and 2.71 eV (458 nm), at 297 K and zero magnetic field. Note that the 1.58 eV Raman spectrum is only shown down to 65 cm^{-1} due to the limitation of our low-frequency filters at this excitation wavelength.

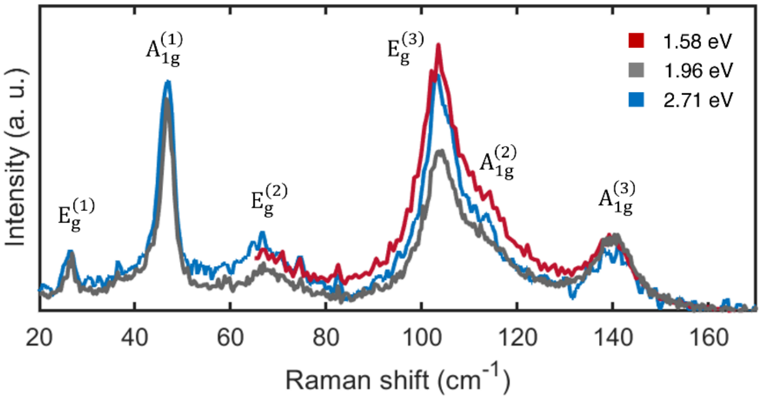

Supplementary Figure 5. Phonon spectra at different laser excitation energies. Raman phonon spectra measured using 1.58 eV (785 nm), 1.96 eV (633 nm), and 2.71 eV (458 nm) laser excitation energies at 297 K and zero magnetic field. Spectra are normalized to the $A_{1g}^{(3)}$ peak intensity.

It is observed that phonon peak intensities indeed change as a function of the laser excitation energy, however, these changes occur across all the observed phonon modes, i. e. the three A_{1g} modes as well as the three E_g modes. It is clear that this result is independent of the choice of normalization. In contrast, the temperature- and field-dependent magnetophononic effects observed in our study are only in the $A_{1g}^{(1)}$ and $A_{1g}^{(2)}$ modes, with negligible changes in the scattering intensities of other modes. Our observations reported in Fig. 1 and Fig. 2 are thus inconsistent with resonant Raman effects.

Furthermore, upon tracking the $A_{1g}^{(2)}$ mode across the PM → AFM transition at 24 K using the 1.58 eV (785 nm) laser excitation, it is found that the Raman scattering intensity exhibits quantitatively the same behavior (see Supplementary Figure 6) as with the 1.96 eV (633 nm) laser excitation (see Fig. 1c) – i. e. the $A_{1g}^{(2)}$ scattering intensity is enhanced by around 35% in the AFM phase. This is additional evidence that the observed phenomenon is inconsistent with resonant Raman effects, wherein different excitation energies would give rise to qualitatively different temperature-dependent intensity changes. It is instead consistent with an effect arising from the AFM order, as in our model of magnetophononic wavemixing.

It is useful to consider the exchange energies involved in various magnetic phase transitions in $MnBi_2Te_4$. The dominant in-plane nearest neighbor exchange coupling is 0.12 meV, whereas the interplanar exchange coupling is an order-of-magnitude weaker[4]. The temperature-driven PM → AFM transition is accompanied by significant magnetic energy changes due to the in-plane ordering of spins, and the large in-plane exchange coupling. On the other hand, the in-plane ordering remains unchanged in the out-of-plane magnetic field-driven AFM → FM transition, with only the interplanar magnetic order being modulated.

The accompanying magnetic energy changes are thus an order of magnitude weaker than in the PM → AFM transition. Hence it is expected that the associated electronic structure changes as a function of out-of-plane magnetic field would also be correspondingly small, minimizing artifacts due to resonant Raman effects. This assertion is validated in our work, where we find that the scattering intensities of the E_g modes and the $A_{1g}^{(3)}$ mode are unchanged as a function of out-of-plane magnetic field within the experimental uncertainty, as outlined in Section S3, allowing us to identify magnetophononic zone-folding in the $A_{1g}^{(1)}$ and $A_{1g}^{(2)}$ peaks. Importantly, the phenomena observed in Fig. 1 and Fig. 2 are correlated not with magnetic order itself, but specifically with AFM order. The zone-boundary phonon intensity vanishes in the FM phase. In fact, as the results in Fig. 2 show, the zone-boundary intensity of the $A_{1g}^{(2)}$ phonon quantitatively tracks the AFM order in both the temperature- and magnetic field-dependent experiments.

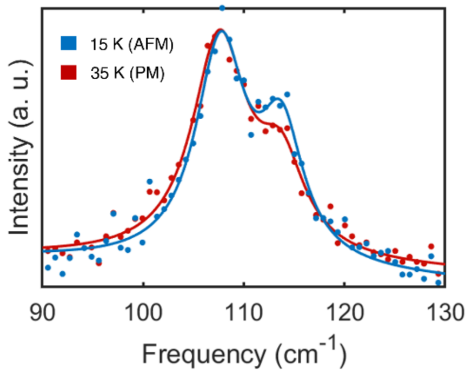

Supplementary Figure 6. Temperature-dependent intensity of $A_{1g}^{(2)}$ mode with 1.58 eV excitation. Raman spectra measured at 15 K and 35 K using a 1.58 eV (785 nm) laser excitation. The dots are experimental datapoints, and the solid lines are fits as outlined in the Methods section.

Based on the above arguments, in order to rule out resonant Raman effects and isolate peak intensity changes due to magnetophononic coupling, it is essential to measure and analyze phonon scattering intensities as a function of both temperature and magnetic field, as carried out in the present work.

Supplementary Note 6: Symmetry of anomalous scattering intensity

Magnetic ordering can potentially give rise to one-magnon and two-magnon resonances in Raman spectra. In order to eliminate the possibility that the anomalous scattering intensities observed in our work (plotted in Fig. 1 and Fig. 2) are due to magnons, we carry out a polarization analysis.

Magnons, by virtue of breaking time-reversal symmetry necessarily have off-diagonal terms in the Raman tensor[5]. In $MnBi_2Te_4$, this is associated with E_g modes, as opposed to A_{1g} modes which are fully symmetric and have only diagonal components. The symmetry associated with Raman scattering intensity can be identified as A_{1g} or E_g using polarized Raman measurements, as in Supplementary Note 1. Here, we focus on the $A_{1g}^{(2)}$ mode. In Supplementary Figure 7, we show Raman spectra obtained below and above the AFM ordering temperature $T_N = 24$ K, corresponding to parallel-polarization, which is sensitive to both A_{1g} and E_g modes, and cross-polarization, which is sensitive only to E_g modes. Our results clearly show that the anomalous scattering intensity overlapped with the $A_{1g}^{(2)}$ phonon in the AFM phase has an A_{1g} symmetry, since it is absent in the cross-polarized channel. This rules out the possibility that it is due to a magnon. It is instead consistent with our interpretation in terms of scattering intensity due to $A_{1g}^{(2)}$ zone-boundary phonons.

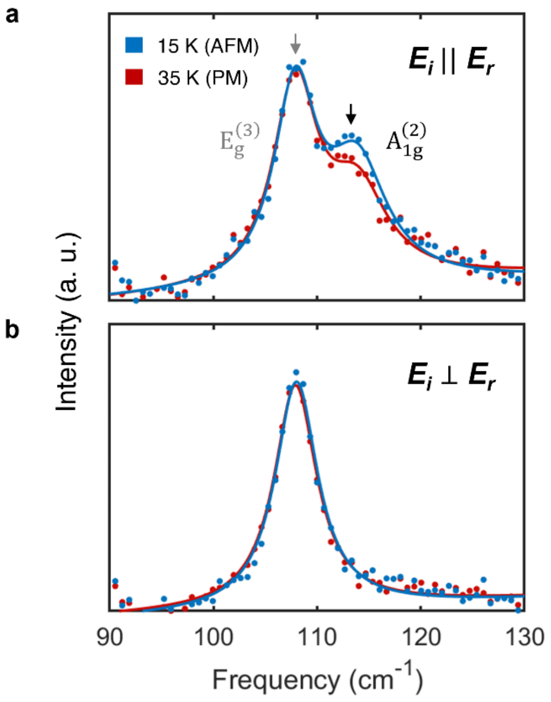

Supplementary Figure 7. Temperature- and polarization-dependent $A_{1g}^{(2)}$ scattering intensity. Raman spectra measured at 15 K and 35 K with parallel- (**a**) and cross-polarization (**b**) of incident and reflected light. The dots are experimental datapoints, and solid lines are fits as outlined in the Methods section.

The above inference is also consistent with magnon dispersions measured using inelastic neutron scattering[4]. The dispersion shows that zone-center magnons are at around 1 meV (~8 cm^{-1}), whereas the highest energy zone-boundary magnons are at 3 meV (~25 cm^{-1}). The low energy of zone-center magnons rules out the possibility of one-magnon resonance interfering with phonon peaks. A two-magnon resonance

may plausibly interfere with the $A_{1g}^{(1)}$ mode at 47 cm^{-1} but would be too low in energy to affect the $A_{1g}^{(2)}$ mode at 115 cm^{-1}, ruling it out as an explanation for the observed phenomena. Two-magnons are also typically associated with a broad continuum of excitations rather than a well-defined peak, a feature that we do not observe in our experiments.

Supplementary Note 7: Pump-probe measurements – Fluence-dependence

The pump-probe measurements outlined in the main text show phonon excitation via a displacive mechanism, where the ultrafast excitation of carriers by the pump pulse shifts the quasiequilibrium ionic coordinates, generating coherent phonons. Here, the amplitude of coherent phonons is directly proportional to the pump-induced carrier density, i. e. the absorbed fluence. In this context, field-dependent optical conductivity changes may influence coherent phonon amplitudes, in addition to the magnetophononic coupling highlighted in the main text. We account for such magnetic-field dependent changes in absorbed fluence by normalizing the pump-probe measurements with respect to the pump-induced carrier density. The carrier density can be tracked by the maximum amplitude of the transient reflectivity trace, which occurs at a time delay of ~0.9 ps. In Fig. 4 of the main text, all the pump-probe traces are normalized with respect to this amplitude.

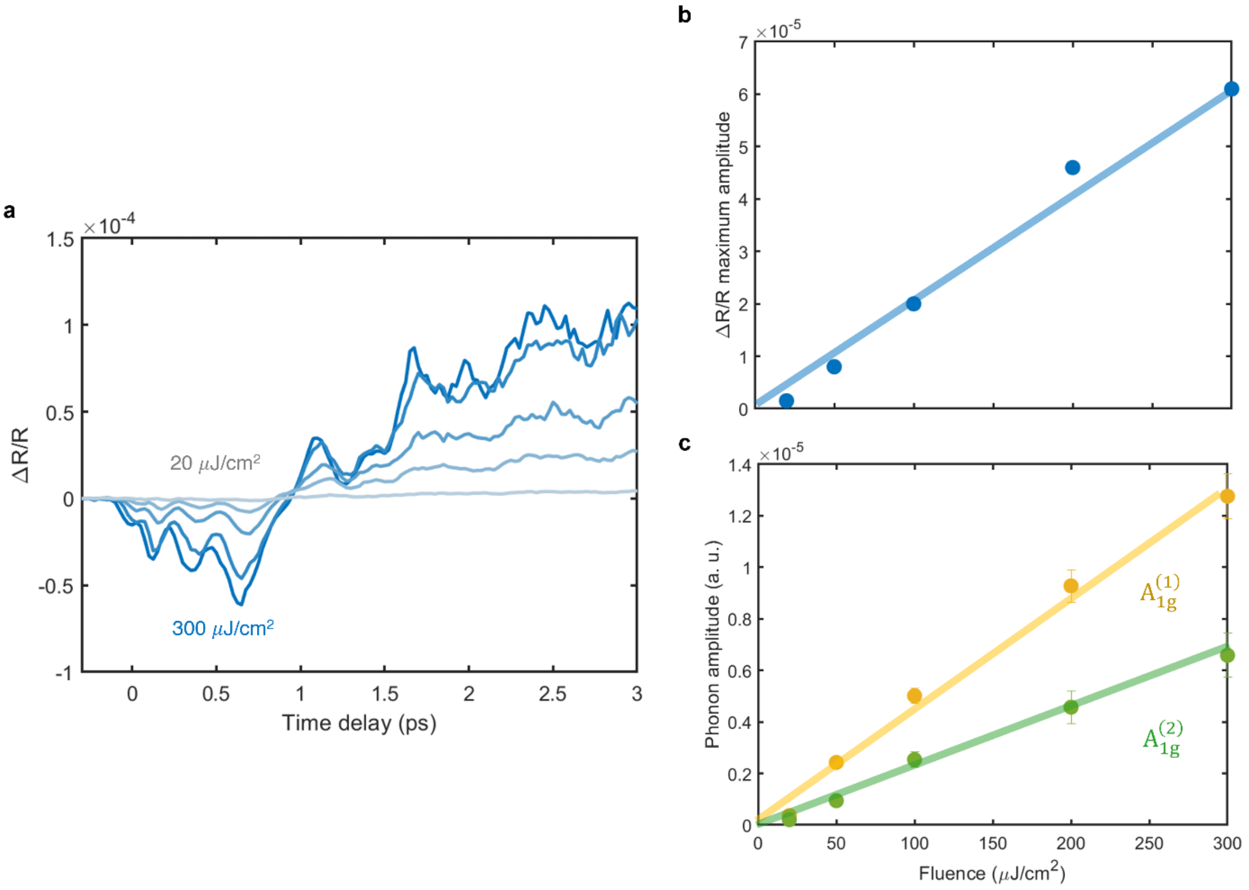

Supplementary Figure 8. Fluence-dependence of coherent phonons. a, Transient reflectivity traces as a function of pump fluence from 20 to 300 μJ/cm^2. **b,** Maximum sub-picosecond amplitude of transient reflectivity as a function of pump fluence, which is a measure of the photoinduced carrier density. **c,** Amplitude of $A_{1g}^{(1)}$ and $A_{1g}^{(2)}$ coherent phonons as a function of pump fluence, extracted using the method outlined in the main text and Methods. The lines in b and c are linear fits. Error bars are standard deviations in fit values.

We verify the validity of this approach by separately measuring the transient reflectivity and coherent phonon amplitudes as a function of pump fluence, shown in Supplementary Figure 8a. The fits in Supplementary Figures 8b and 8c, carried out as outlined in the Methods, show that both the maximum

transient reflectivity (which tracks the absorbed fluence and photoinduced carrier density) as well as the unnormalized coherent phonon amplitudes scale linearly with fluence, confirming that the maximum amplitude of the transient reflectivity trace indeed tracks the carrier density, validating the normalization procedure used in the main text.

Finally, we note that outside of magnetic-field dependent changes in absorbed fluence, there may potentially be additional magnetodielectric effects that change the electron-phonon interactions and thus the Raman susceptibility, which can affect coherent phonon generation. To lowest order, such changes may be phenomenologically described by a magnetodielectric effect of the form $\chi_e = \chi_e^{(0)} + \gamma M^2$, where χ_e is the electrical susceptibility, γ is magnetodielectric coefficient, and M is the net magnetization. Below, we explore possible changes to electron-phonon interaction due to such a magnetodielectric effect. In the interest of conceptual clarity, we consider a simple M-H dependence $M = \chi_m H$, where χ_m is the magnetic susceptibility and H is the external magnetic field, the Raman susceptibility of a phonon (which determines the coherent phonon amplitude via a Raman-like displacive excitation) can then be written as $\frac{d\chi_e}{du} = \frac{d\chi_e^{(0)}}{du} + H^2\left(2\gamma\chi_m \frac{d\chi_m}{du} + \chi_m^2 \frac{d\gamma}{du}\right)$. The expression in parenthesis determines the change in Raman susceptibility due to the magnetic field, where $\frac{d\chi_m}{du}$ and $\frac{d\gamma}{du}$ are the phonon modulation of the magnetic susceptibility and magnetodielectric coupling coefficient, respectively. Such a field-dependent change in the coherent phonon amplitude would then be a form of indirect magnetophononic coupling. Based on our current pump-probe experimental data, we cannot completely rule out that such indirect magnetophononic effects also have a contribution, in addition to the direct magnetophononic effects highlighted in our manuscript.

Supplementary Note 8: Debye-Waller effect in ultrafast electron diffraction

Optical pump-probe experiments are typically initiated by ultrafast optical (pump) pulses which generate photo-excited carriers (electrons and holes) – which, after thermalizing, decay through the generation of lattice (and spin) excitations. The lattice excitations result in a disordering of the lattice. Ultrafast electron diffraction is a direct probe of this pump-induced lattice disorder, through the transient Debye-Waller effect.

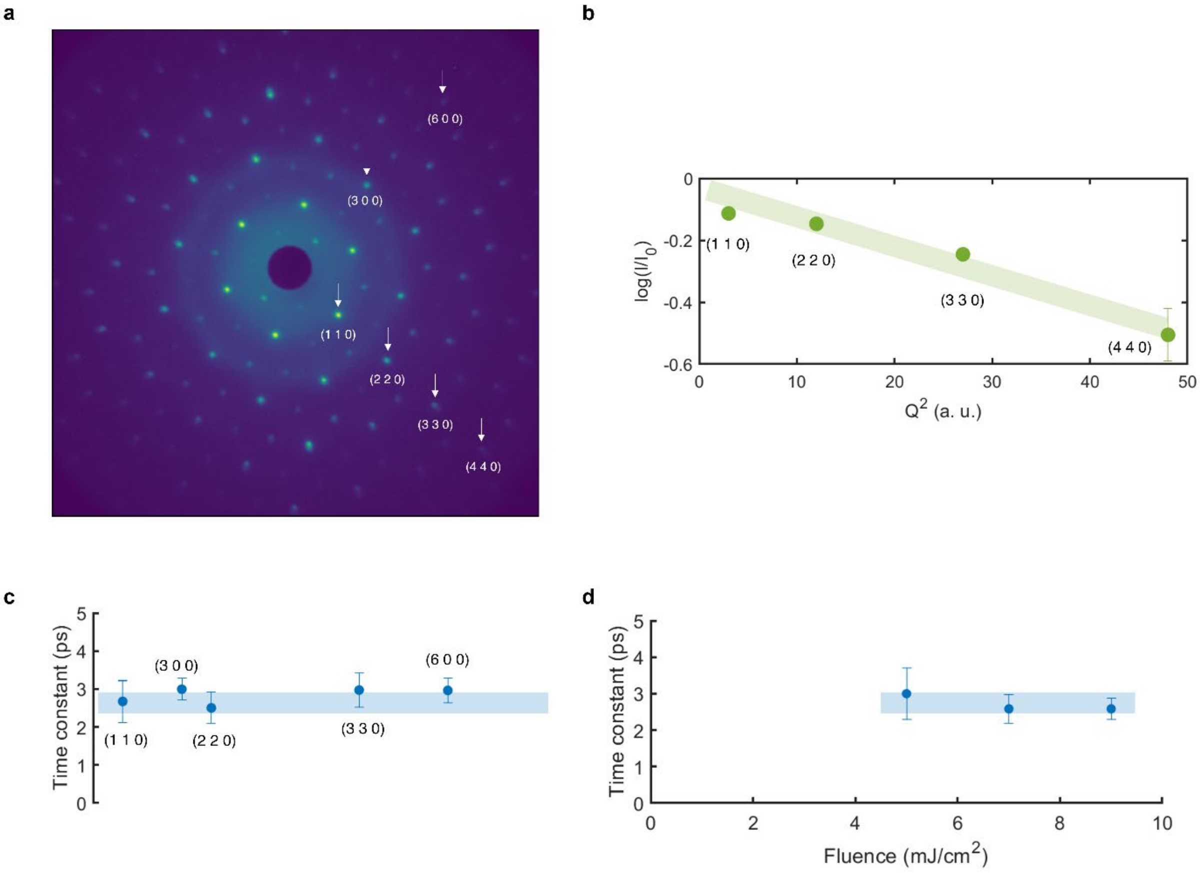

Supplementary Figure 9. Transient Debye-Waller effect. a, Static electron diffraction image, with the (n n 0) family of peaks labeled. **b,** Transient Bragg reflection intensity obtained in the $t \to \infty$ limit from the exponential decay fit, plotted as a function of $\boldsymbol{Q}$. The green line is a guide to the eye. **c,** The exponential decay constant of the transient intensity of various Bragg peaks. **d,** The exponential decay constant of the (2 2 0) peak as a function of pump fluence. Error bars are standard deviations of fit values. The blue lines are guides to the eye.

In general, the intensity of a Bragg reflection in an electron diffraction experiment is given by

$$I_0(Q) = \sum_j f_j(Q) \exp\left(-2\pi^2 B_j Q^2\right) \exp\left(-i2\pi Q.r_j\right), \qquad (5)$$

where the summation is over atoms in the unit cell (indexed by j), Q is the scattering wavevector, f_j is the atomic structure factor for electron diffraction for atom j, B_j is the isotropic Debye-Waller factor for atom j, and r_j is the position of atom j in the unit cell. The Debye-Waller factor is given by $B_j = \langle u_j^2 \rangle$, which is the root-mean-square displacement of atom j about its mean position. A representative static diffraction pattern from a ~100 nm flake of $MnBi_2Te_4$ oriented along the (0 0 1) crystallographic direction is shown in Supplementary Figure 9a.

Pump-induced lattice disorder increases B_j, i. e. $B_j \rightarrow B_j + \Delta B_j$, thus generally resulting in a decrease in the transient Bragg reflection intensities. We can define an effective transient Debye-Waller factor ΔB_{eff} as follows –

$$I(Q) = \sum_j f_j(Q) \exp\left(-2\pi^2 \left(B_j + \Delta B_j\right) Q^2\right) \exp\left(-i2\pi Q.r_j\right)$$

$$= \exp\left(-2\pi^2 \Delta B_{eff} Q^2\right) I_0(Q), \quad (6)$$

The transient Bragg reflection intensity $I(Q)$ is thus a quantitative measure of the pump-induced lattice disorder, $\Delta B_{eff} = \Delta\langle u_{eff}^2\rangle$. The Debye-Waller effect for a given family of Bragg reflections scales with Q, such that $\log\left(\frac{I}{I_0}\right) \propto -Q^2$.

In Supplementary Figure 9b, we plot the transient intensity $\log\left(\frac{I}{I_0}\right)$ of the (*n n 0*) family of Bragg reflections at the $t \rightarrow \infty$ limit from the exponential decay fit (see Methods), as a function of Q^2. The linear dependence confirms that the observed evolution of transient Bragg intensities is due to a Debye-Waller effect via pump-induced lattice disorder.

The time constants of the transient intensities of various Bragg peaks, obtained from exponential decay fits, are shown in Supplementary Figure 9c. Within the experimental uncertainty, the time constant is uniform across different peaks.

In the main text, we use the transient Debye-Waller time constant from our UED measurements to establish the timescale of lattice thermalization. However, the optical pump-probe measurements reported in the main text use a much lower fluence, of 0.1 mJ/cm^2, as opposed to 7 mJ/cm^2 used in the UED measurements reported above. In this context, we report the thermalization time constants from our UED measurements as a function of fluence, in Supplementary Figure 9d. The time constants are largely unchanged from 5 to 9 mJ/cm^2, with a slight increase at lower fluences.

Such a behavior is consistent with increased phonon-phonon scattering at higher fluences[6]. It is expected then that the thermalization time constant at the low fluences used in our optical measurements, with their correspondingly lower phonon populations, would likely be even higher than that extracted from the UED measurements; i. e. the UED time constant sets a lower bound for the phonon thermalization time. This supports our assertion that phonon subsystem remains in a nonequilibrium state through the entire time delay range measured in our study.

1. Köpf, M., Ebad-Allah, J., Lee, S. H., Mao, Z. Q. & Kuntscher, C. A. Influence of magnetic ordering on the optical response of the antiferromagnetic topological insulator MnBi2Te4. *Phys. Rev. B* **102**, 165139 (2020).

2. Baltensperger, W. & Helman, J. S. Influence of magnetic order in insulators on the optical phonon frequency. *Helv. Phys. Acta* **41**, 668–673 (1968).

3. Choe, J. *et al.* Electron–Phonon and Spin–Lattice Coupling in Atomically Thin Layers of MnBi 2 Te 4. *Nano Lett.* **21**, 6139–6145 (2021).

4. Li, B. *et al.* Competing Magnetic Interactions in the Antiferromagnetic Topological Insulator MnBi2Te4. *Phys. Rev. Lett.* **124**, 167204 (2020).

5. Antoinette, J. & Rohit, P. *Optical techniques for solid state materials characterization*.

6. Konstantinova, T. *et al.* Nonequilibrium electron and lattice dynamics of strongly correlated Bi 2 Sr 2 CaCu 2 O 8+δ single crystals. *Sci. Adv.* **4**, eaap7427 (2018).
